# Typical and atypical CT chest imaging findings of novel coronavirus 19 (COVID-19) in correlation with clinical data: impact on the need to ICU admission, ventilation and mortality

**DOI:** 10.1186/s43055-020-00339-3

**Published:** 2020-11-11

**Authors:** Doaa M. Emara, Nagy N. Naguib, M. A. Moustafa, Salma M. Ali, Amr Magdi El Abd

**Affiliations:** 1 Department of Radiodiagnosis, Faculty of Medicine, University of Alexandria, Alexandria, Egypt; 2 Department of Radiodiagnosis, AMEOS Hospital Halberstadt, Halberstadt, Germany; 3 Department of Anaesthesia and Surgical Intensive Care, Faculty of Medicine, University of Alexandria, Alexandria, Egypt; 4 Department of Community Medicine, Faculty of Medicine, University of Alexandria, Alexandria, Egypt

**Keywords:** Coronavirus-PCR, COVID-19, Chest CT scan, Outbreak-pandemic, Pneumonia

## Abstract

**Background:**

The aim of this study was to highlight the typical and atypical chest CT imaging features at first presentation in 120 patients who were proved to be COVID-19 by PCR and to correlate these findings with the need for ICU admission, ventilation, and mortality. We retrospectively included 120 patients 71 males (59.2%) and 49 females (40.8%) with a mean age of 47.2 ± 14.4 years. Patients subjected to clinical assessment, CBC, PCR for COVID-19, and non-contrast CT chest at first presentation. Typical and atypical imaging findings were reported and correlated with the clinical findings of the patients, the need for ICU admission, ventilation, and mortality.

**Results:**

Clinically, fever was seen in 112 patients followed by dry cough in 108 patients and malaise in 35 patients. The final outcome was complete recovery in 113 cases and death in 7 cases. Typical CT findings included bilateral peripheral ground-glass opacities (GGO) in 74.7%, multilobar affection in 92.5% while atypical findings such as homogeneous consolidation, pleural effusion, mediastinal lymphadenopathy, and single lobar affection were found in 13.4, 5, 6.7, and 7.5% respectively. A statistically significant association between the presence of white lung, pleural effusion, peripheral GGO, and the need for ICU admission as well as mechanical ventilation was noted. The death was significantly higher among elderly patients; however, no significance was found between the imaging features and mortality.

**Conclusion:**

CT features at first presentation can predict the need for ICU admission and the need for ventilation but cannot predict the mortality outcome of the patients.

## Key points

1. MDCT plays an important role in the diagnosis of COVID-19 in correlation with clinico-laboratory data.
2. Typical and atypical CT findings in COVID-19 infection were found.
3. CT imaging findings can predict the patient’s need for ICU admission and mechanical ventilation but not patient death.

## Background

Coronavirus is RNA enveloped non-segmental virus characterized by the presence of spikes on its surface; it causes respiratory tract infection of variable degrees up to acute respiratory distress syndrome (ARDS) and death [1].

In December 2019, an outbreak of pneumonia occurred in Wuhan, China; first, it was of unknown etiology and then it was proven to be a viral infection with the novel coronavirus. In the beginning, it was of zoonotic transmission through direct contact with a local fish market; however, the rapid direct person-to-person transmission was found resulting in a very rapid increase in the number of infected people per day reaching thousands and spreading worldwide. On 11 March 2020, the WHO declared the coronavirus outbreak a pandemic [2, 3].

For diagnosis of COVID-19, PCR is used nowadays with a sensitivity reaching 70% and may increase according to the virus load in the specimen either nasopharyngeal or oropharyngeal swab and even bronchoalveolar lavage. Also, CBC changes with lymphopenia have been described [4].

The main concern about the rapid spread of the virus leading to an extensive increase in the number of simultaneously infected patients with rapid exhaustion of the health facilities and in extreme cases selecting which patients to treat and give maximum health support based on their theoretical expected outcome. Different studies showed that the CT imaging findings, as viral pneumonia, are sensitive in the diagnosis of COVID positive cases and even may precede the laboratory findings [3, 5]. Hence, it might be beneficial to use the CT findings to provide an objective tool in stratifying the patients to point out patients who will need ICU admission, those who will need mechanical ventilation, and those who have a higher mortality expectancy. Based on this, the current study was performed in order to study the spectrum of CT imaging findings in 120 COVID-19 proven patients at their first presentation, to classify these findings to typical and atypical findings, and to correlate these findings and the clinical findings with the patients’ outcome and its impact on health system namely the need for ICU admission and mechanical ventilation.

## Methods

The current retrospective study was approved by the Ethical Committee of our university hospital. We included 120 COVID-19 positive cases from May 2020 to July 2020 and revised their clinical, laboratory, and imaging findings.

The inclusion criteria included:

1. Patients with proven diagnosis of COVID-19 clinically and with positive PCR.
2. Available CT examination (first CT at the time of presentation).

The exclusion criteria included:

1. Patients with no available PCR test confirming the diagnosis of COVID-19
2. Patients with previous known structural lung diseases to avoid overlap from previous lung diseases.
3. Patients with no CT examination on first presentation available

All patients were subjected to the followings:

- Clinical assessment regarding the presence of fever, dry cough, malaise, shortness of breath.
- Laboratory investigation: including CBC and PCR of novel coronavirus (COVID-19).
- Non-contrast CT of the chest at first presentation and later as clinically indicated.

### Imaging technique

All CT examinations were done using Siemens Perspective 64-slices scanner. The CT protocol was as follows: axial scans craniocaudal direction, breath-hold at full inspiration with tube voltage 130 kV, mAs 102, slice thickness 1 mm, pitch 1.2, and rotation time 0.6 s. The mediastinal and lung windows were assessed using the dedicated workstation.

### Imaging interpretation

All examinations were assessed by two radiologists with more than 17 and 20 years of experience in CT chest in consensus. Owing to the current pandemic of the known clinical context, the readers could not be blinded to the fact that the patients might be COVID-19 positive. However, they were blinded to the fact that all presented images were from proven COVID-19 positive patients and not due to other causes of pneumonia. The readers were only informed about the clinical presentation of the patients with COVID-19 as one of the possible diagnoses.

The radiologists were requested to assess the images regarding the following parameters:

- Ground-glass opacities, crazy-paving lesions, and their distribution at both lungs. (As signs of severity in CT depends on degree or percent of the affected lung parenchyma)
- Associated subpleural thickening, pulmonary nodules, cavitary lesions, consolidation whether homogenous or heterogeneous on top of ground-glass densities.
- Pleural effusion (as the presence of pleural effusion indicates more severe condition, not only parenchymal affection and usually has an impact on patient’s clinical findings and progression of dyspnea*),* mediastinal, and hilar lymphadenopathy.

### Study design and statistical analysis

We studied the association between the CT features and clinical data among our cases, its impact on the health system including ICU admission, the need for mechanical ventilation, and mortality. In addition, typical and atypical CT findings were correlated with the clinical presentation of the patients.

Frequencies (percent) and means (± SD) were used to summarize categorical and continuous variables, respectively. Associations were conducted using the Mann-Whitney *U* test and chi-square tests. Univariate logistic regression models were used to evaluate the association of outcome and other variables: age, gender, clinical picture as shortness of breath, tachypnea and headache, the need for ventilation, and severe radiological signs. The interaction between outcome status and different independent variables in relation to the dependent variable was tested. Backward binary logistic regression was used to select statistically significant variables, and possible confounding factors associated with outcome were included in the final model. Analyses were done by the Statistical Package for Social Sciences (version 24). A *p* value <0.05 level was used as the cut-off value for statistical significance.

## Results

### The studied population

Among the studied 120 patients, 71 were males (59.2%) and 49 were females (40.8%) with mean age 47.2 ± 14.4 years (range 12.0–76.0 years).

All 120 COVID-19 had positive PCR results and lymphopenia (lymphocytes <10% of the total leukocytic count) was found in 112 cases (93.3%).

### Clinical data

The main presentation among the studied cases was fever (temperature > 37.5 at the time of examination or history of fever during the past 48 h) as it was found in 112 patients followed by dry cough in 108 patients and malaise in 35 patients. Twenty-five patients were admitted to the ICU without the need for mechanical ventilation; 13 patients presented with severe clinical symptoms progressed to ARDS-like and have been admitted to the ICU and placed on a ventilator (Table 1). The final outcome was complete recovery in 113 cases and death in 7 cases.

**Table 1:** Summarize the clinical data of the studied cases

Clinical picture^a^	***n***	%
Fever^b^	112	93.3
Nasal congestion	12	10.0
Sore throat	19	15.8
Cough	108	90.0
Shortness of breath^b^	11	9.2
Tachypnea^b^	7	5.8
Headache	19	15.8
Malaise	35	29.2
Diarrhea	8	6.7

^a^Cells are not mutually exclusive

^b^Fever: temperature > 37.5 at the time of examination or history of fever during the past 48 h. Tachypnea: respiratory rate > 30/min. Shortness of breath: intense tightness in the chest or air hunger

The patients who died were older (55.6 ± 23.3 years old) than those who recovered (46.7 ± 13.6 years old) and this age difference was statistically significant, (*p* = 0.05).

Regarding the clinical picture, more than half of patients who died (57.1%) suffered from shortness of breath or tachypnea compared with a small percent among those who recovered (6.2 and 2.7%, respectively) with a highly statistically significant association. Moreover, 71.4% of those who died suffered from headache compared with only 12.4% among recovered, (*p* = <0.001) (Table 2).

**Table 2:** Association between outcome with age and the significant clinical features

	Outcome				Unadjusted odds ratio CI(UL-LL)	Test of significance (***p*** value)
	Recovery (***n*** = 113)		Death (***n*** = 7)			
	***n***	%	***N***	%		
• **Age**						
Mean ± SD	46.7 ± 13.6		55.6 ± 23.3		–	**Mann–Whitney** *Z* = 1.935 *p* = 0.05*
Median	48.0		67.0			
Min.-max.	15–76		12–73			
• **Shortness of breath**						
No	106	93.8	3	42.9	20.2 (3.7–108.4)	**Chi-square test** *X*^*2*^ = 20.549 *p* = <0.001*
Yes	7	6.2	4	57.1		
• **Tachypnea**						
No	110	97.3	3	42.9	48.9 (7.4–322.3)	**Chi-square test** *X*^*2*^ = 35.627 *p* = <0.001*
Yes	3	2.7	4	57.1		
• **Headache**						
No	99	87.6	2	28.6	17.7 (3.1–99.9)	**Chi-square test** *X*^*2*^ = 17.051 *p* = <0.001*
Yes	14	12.4	5	71.4		

*Significant at *p* ≤ 0.05

### CT chest imaging findings

The radiological signs among the studied cases are summarized in Table 3.

**Table 3:** The radiological signs of the studied cases

Radiological findings^a^	***n***	%
Ground glass opacity	120	100.0
o Multilobes	111	92.5
⇒ Peripheral	83	74.7
⇒ Central and Peripheral	20	18.1
⇒ White lung	8	7.2
o Single lobe	9	7.5
Consolidation	16	13.3
o Heterogenous	13	11.7
o Homogenous	3	13.4
Crazy paving	39	32.5
Sub-pleural atelectasis	9	7.5
Tree in bud nodules	9	7.5
Pleural effusion	6	5.0
Lymph nodes	8	6.7
CT severity signs^b^	21	17.5

^a^Cells are not mutually exclusive

^b^Signs of severity by CT included multiple bilateral peripheral and central GGO, white lung and pleural effusion

The typical CT findings were peripheral bilateral ground-glass opacities, multilobar affection, while the atypical CT findings including, peripheral and central multiple bilateral ground-glass opacities, consolidation, white lung, single-lobar affection, presence of pleural effusion, and mediastinal lymphadenopathy (Table 4).

**Table 4:** Summarize the typical and atypical CT findings in the studied COVID-19 patients

Typical findings	Number	%
Peripheral ground-glass patches	83	74.7
Multilobar affection	111	92.5
**Atypical findings**		
Central and peripheral ground-glass patches	20	18.1
White lung	8	7.2
Consolidation mainly if homogenous	3	13.4
Single lobar affection	9	7.5
Sub-pleural atelectasis	9	7.5
Pleural effusion	6	5.0
Enlarged mediastinal lymph nodes	8	6.7

**Fig. 1:**
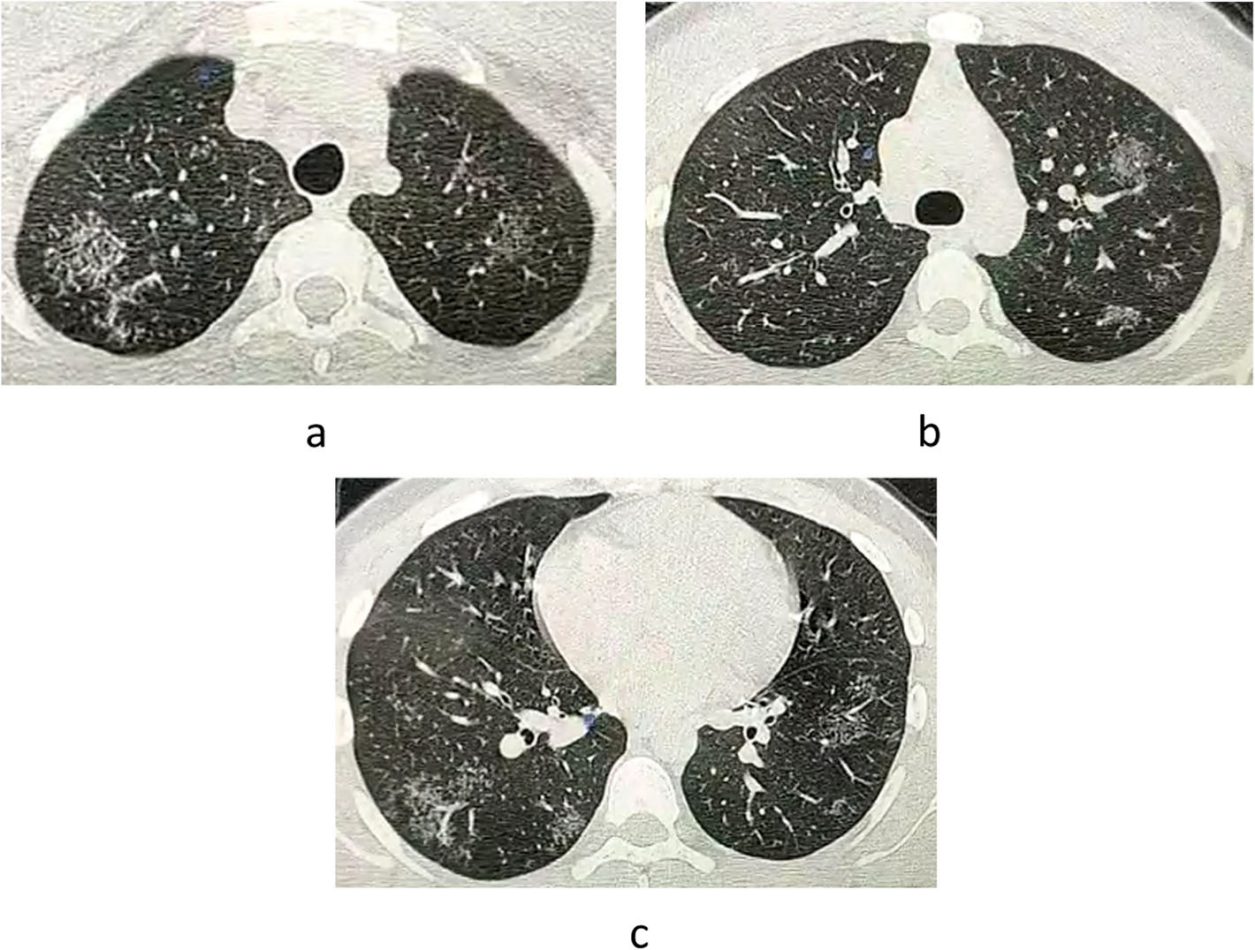
Axial CT lung window in 52-year-old male patient showed well-defined multiple GGO scattered at both lungs at different levels of peripheral distribution. Typical CT features of COVID-19

The interpretation of the studied cases by the reading radiologists revealed 86 cases were diagnosed radiologically as COVID-19 by classic typical bilateral GGO (Fig. 1). In the remaining 34 cases with non-classic atypical CT findings, the differential diagnosis of other causes of chest infection was included in addition to COVID-19.

### Correlation between CT severity signs and the clinical presentation of the patients

From the studied 120 patients, we classified CT signs of severity as bilateral diffuse multiple multilobar peripheral and central ground-glass patches, presence of white lung, and development of pleural effusion. The only CT features that were statistically significantly associated with severe shortness of breath and tachypnea were the presence of pleural effusion (*p* < 0.001 and *p* = 0.003 respectively). The presence of white lung and multiple peripheral and central GGO did not statistically significantly correlate with the presence of shortness of breath and tachypnea (*p* = 0.735 and 0.466 for white lung correlation) and (*p* = 0.887 and 0.223 for GGO) respectively.

### Imaging findings and the need for ICU admission and mechanical ventilation

**Fig. 2:**
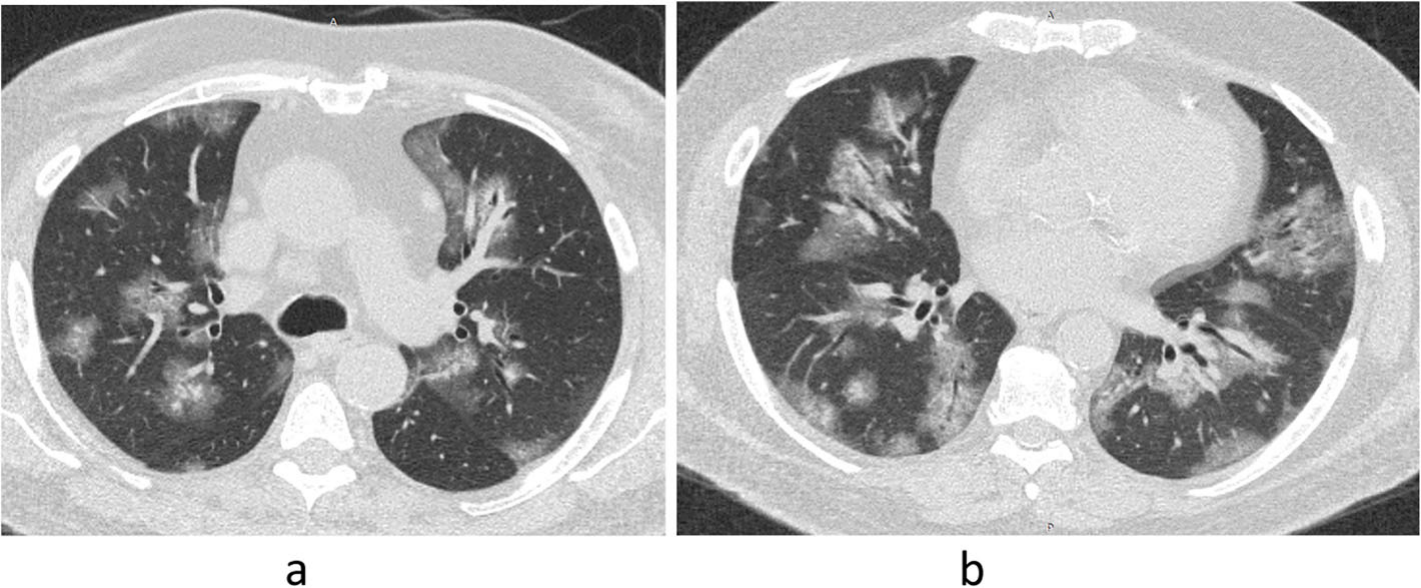
A 65-year-old male patient: (**a**, **b**) axial CT lung window at different levels showed multiple scattered irregular patchy ground-glass lesions with crazy-paving pattern

**Fig. 3:**
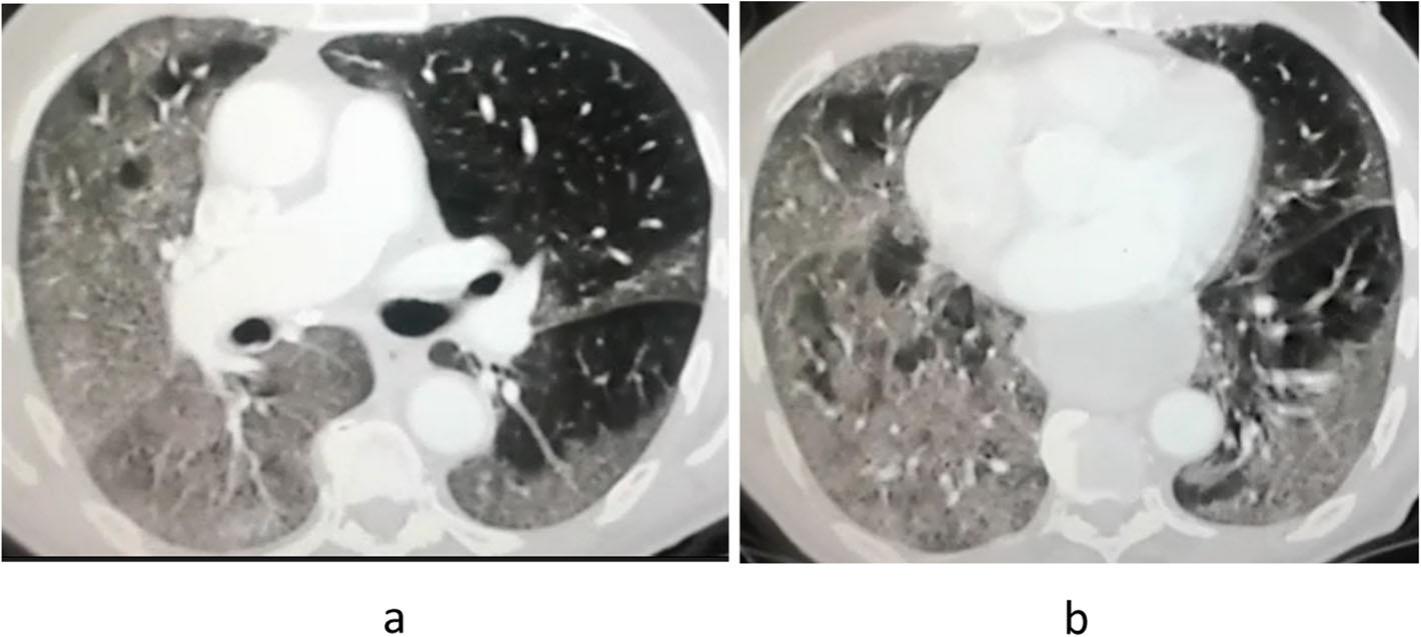
**a**, **b** Axial CT chest lung window in a 67-year-old male patient showed a diffuse homogenous ground-glass density of both lungs, more evident on the right side (white lung). Atypical CT features of COVID-19; clinically, this patient suffered from severe clinical symptoms progressed to ARDS and then mechanically ventilated

**Fig. 4:**
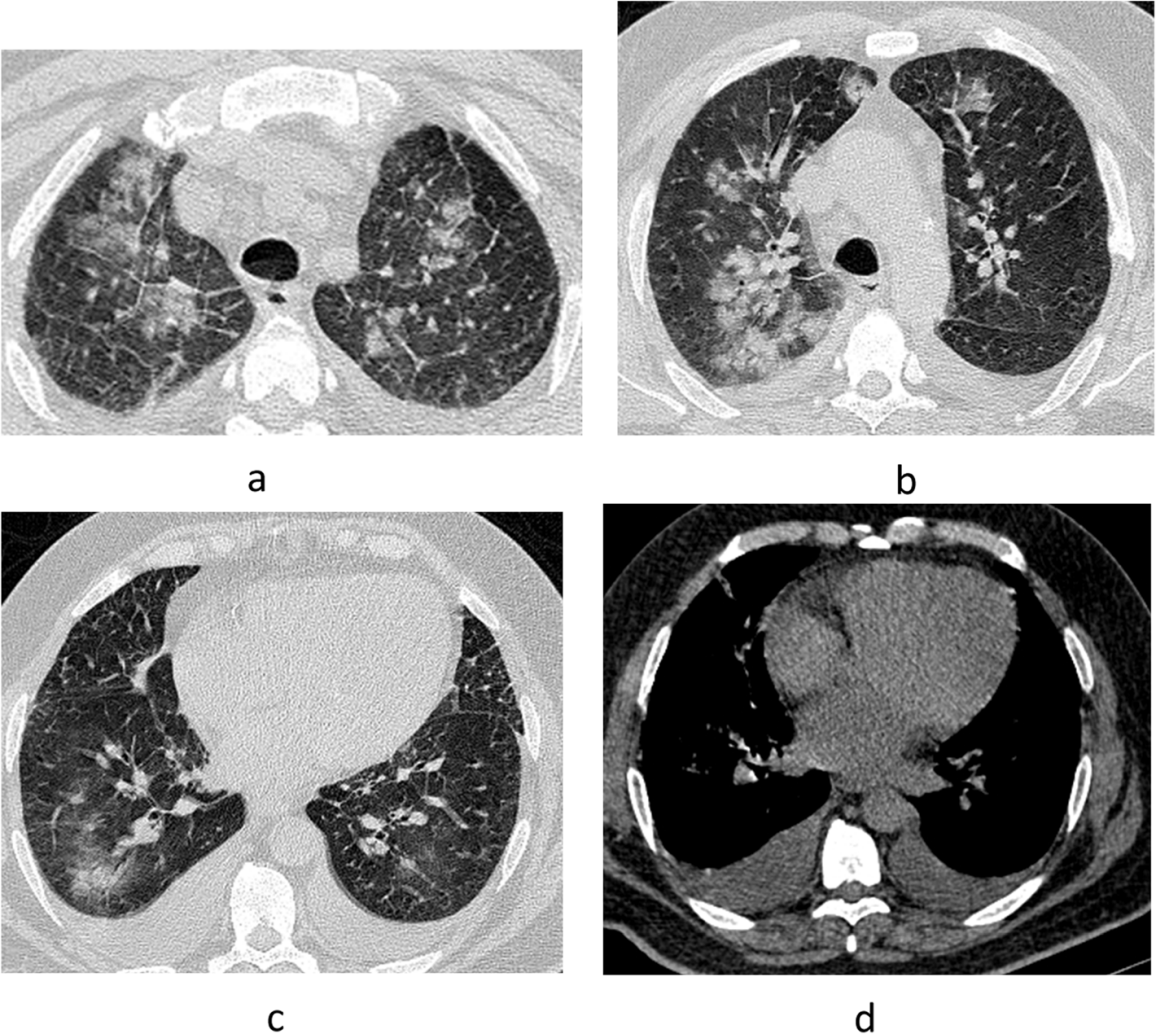
Male patient 58 years (**a**–**c**) axial CT lung window showing multiple confluent GGO scattered at both lungs involving the whole lobes; this is associated with bilateral mild to moderate pleural effusion in axial CT mediastinal window (**d**)

From our cases, we found a strong association with a statistically significant difference between the presence of multiple multilobar peripheral GGO, white lung, and pleural effusion in CT and the need for ICU admission and mechanical ventilation (Figs. 2, 3, 4). Tables 5 and 6 summarize the correlation between the clinical and CT features and the need for ICU admission and mechanical ventilation.

**Table 5:** Association between radiological findings with the need for ventilation

	Ventilation		Chi-square test (***p*** value)
	No (***n*** = 107)	Yes (***n*** = 13)	
	%(***n***)	%(***n***)	
**Radiological findings**			
CT severity signs	10.3(11)	76.9(10)	*X*^*2*^ = 35.658 *p* = <0.001*
Ground glass opacity			
o Multilobes	92.5(99)	92.3(12)	*X*^*2*^ = 0.001 *p* = 0.978
⇒ Peripheral	73.8(79)	30.8(4)	*X*^*2*^ = 10.079 *p* = 0.001*
⇒ Central and peripheral	16.8(18)	15.4(2)	*X*^*2*^ = 0.017 *p* = 0.895
⇒ White lung	1.9(2)	46.2(6)	*X*^*2*^ = 36.535 *p* = <0.001*
o Single lobe	7.5(8)	7.7(1)	*X*^*2*^ = 0.001 *p* = 0.978
Consolidation	14.0(15)	7.7(1)	*X*^*2*^ = 0.401 *p* = 0.526
o Heterogenous	11.2(12)	7.7(1)	*X*^*2*^ = 0.149 *P* = 0.700
o Homogenous	2.8(3)	0.0(0)	*X*^*2*^ = 0.374 *p* = 0.541
Crazy paving	33.6(36)	23.1(3)	*X*^*2*^ = 0.590 *p* = 0.442
Atelectatic bands	7.5(8)	7.7(1)	*X*^*2*^ = 0.001 *p* = 0.978
Tree in bud nodules	7.5(8)	7.7(1)	*X*^*2*^ = 0.001 *p* = 0.978
Pleural effusion	2.8(3)	23.1(3)	*X*^*2*^ = 10.030 *p* = 0.002*
Lymph nodes	7.5(8)	0.0(0)	*X*^*2*^ = 1.041 *p* = 0.307

*Significant at *p* ≤ 0.05

**Table 6:** Association between radiological findings with the need for ICU admission

	ICU admission		Chi-square test (***p*** value)
	No (***n*** = 95)	Yes (***n*** = 25)	
	%(***n***)	%(***n***)	
**Radiological findings**			
CT severity signs	2.1(2)	76.0(19)	*X*^*2*^ = 74.854 *p* = <0.001*
Ground glass opacity			
o Multilobes	91.6(87)	96.0(24)	*X*^*2*^ = 0.558 *p* = 0.455
⇒ Peripheral	73.7(70)	52.0(13)	*X*^*2*^ = 4.364 *p* = 0.037*
⇒ Central and Peripheral	16.8(16)	16.0(4)	*X*^*2*^ = 0.010 *p* = 0.920
⇒ White lung	1.1(1)	28.0(7)	*X*^*2*^ = 23.098 *p* = <0.001*
o Single lobe	8.4(8)	4.0(1)	*X*^*2*^ = 0.558 *p* = 0.455
Consolidation	11.6(11)	20.0(5)	*X*^*2*^ = 1.215 *p* = 0.270
o Heterogenous	8.4(8)	20.0(5)	*X*^*2*^ = 2.747 *p* = 0.097
o Homogenous	3.2(3)	0.0(0)	*X*^*2*^ = 0.810 *p* = 0.368
Crazy paving	33.7(32)	28.0(7)	*X*^*2*^ = 0.291 *p* = 0.589
Atelectatic bands	8.4(8)	4.0(1)	*X*^*2*^ = 0.558 *p* = 0.455
Tree in bud nodules	6.3(6)	12.0(3)	*X*^*2*^ = 0.922 *p* = 0.337
Pleural effusion	1.1(1)	20.0(5)	*X*^*2*^ = 14.958 *p* = <0.001*
Lymph nodes	6.3(6)	8.0(2)	*X*^*2*^ = 0.090 *p* = 0.764

*Significant at *p* ≤ 0.05

### Imaging findings and patient outcome

None of the CT imaging features correlated significantly with patients’ death (all *p* > 0.05) suggesting that even patients with severe imaging features at first presentation might not necessarily die (Table 7).

**Table 7:** Association between radiological findings with death outcome

	Death		Chi-square test (***p*** value)
	No (***n*** = 113)	Yes (***n*** = 7)	
	%(***n***)	%(***n***)	
**Radiological findings**			
CT severity signs	16.8(19)	28.6(2)	*X*^*2*^ = 0.631 *p* = 0.427
Ground glass opacity			
o Multilobes	92.9(105)	85.7(6)	*X*^*2*^ = 0.493 *p* = 0.482
⇒ Peripheral	69.0(78)	71.4(5)	*X*^*2*^ = 0.018 *p* = 0.894
⇒ Central and Peripheral	17.7(20)	0.0(0)	*X*^*2*^ = 1.487 *p* = 0.223
⇒ White lung	6.2(7)	14.3(1)	*X*^*2*^ = 0.694 *p* = 0.405
o Single lobe	7.1(8)	14.3(1)	*X*^*2*^ = 0.493 *p* = 0.482
Consolidation	13.3(15)	14.3(1)	*X*^*2*^ = 0.006 *p* = 0.939
o Heterogenous	10.6(12)	14.3(1)	*X*^*2*^ = 0.092 *p* = 0.762
o Homogenous	2.7(3)	0.0(0)	*X*^*2*^ = 0.191 *p* = 0.662
Crazy paving	31.0(35)	57.1(4)	*X*^*2*^ = 2.058 *p* = 0.151
Atelectatic bands	7.1(8)	14.3(1)	*X*^*2*^ = 0.493 *p* = 0.482
Tree in bud nodules	8.0(9)	0.0(0)	*X*^*2*^ = 0.603 *p* = 0.438
Pleural effusion	4.4(5)	14.3(1)	*X*^*2*^ = 1.349 *p* = 0.245
Lymph nodes	7.1(8)	0.0(0)	*X*^*2*^ = 0.531 *p* = 0.466

*Significant at *p* ≤ 0.05

## Discussion

The COVID-19 pandemic of atypical pneumonia leads to health emergency problems all over the world which is more or less similar to SARS in 2003 and MERS in 2012; all these outbreaks occurred due to virus belonging to the family coronaviridae [6].

Fever and cough were the most common symptoms in patients with COVID-19. The incidence of other upper and severe lower respiratory tract symptoms as well as headache was ranging between 10 and 20%. Diarrhea occurred infrequently and was not encountered in our patient collective at first presentation. Malaise approached 30% of our patients. Thirteen out of 120 patients required mechanical ventilation. This may be consistent with several previous studies documenting that the main target cells for the virus are those located in the lower respiratory tract [7].

Early studies showed that the main diagnostic CT imaging features of COVID-19 are bilateral multilobar peripheral GGO [6, 8]. The presence of peripheral and central GGO, as well as single lobe affection, consolidation, pleural effusion, and mediastinal lymphadenopathy, are not matching with COVID-19; however, later on, after an increased number of diseased cases worldwide was reported, these CT imaging features had been found in some COVID-19 positive patients although it is uncommon [2, 9]. In the current study, we also confirmed that peripheral GGO scattered at both lungs is the most common finding as it was found in 83 cases (74.7%) while the other findings were uncommonly found in a much smaller number of cases. This also was in agreement with Adam et al. [3] and Yan et al. [5]

Song et al. [2] reported that GGO mainly of peripheral distribution in multilobes is the most common imaging feature in COVID-19 positive patients and may be associated with interlobar and interlobular thickening, even consolidation may be present in less frequency. The results of our cases were similar to these findings.

In clinically severe cases, we detected the presence of multiple bilateral central and peripheral GGO in 20 cases, white lung in eight cases, and pleural effusion in six cases. Zu et al. [4] also described these findings as imaging features in severely affected patients. Also, we reported a few cases with pleural effusion (six cases) and mediastinal lymphadenopathy (eight cases) which was also confirmed as atypical findings in other studies [5].

From the results of our cases, we noticed the added value of CT chest imaging with clinical data to diagnose COVID-19 patients and this is helpful in situations such as shortage of test kits or false-negative results; this was in agreement with Wenjing et al. [10] who also concluded the importance of imaging in the diagnosis of COVID-19 patients.

After increasing the number of affected patients worldwide and unfortunately the health systems in most countries cannot cope with this pandemic, most of the studies should look for the impact of the disease course on the outcome and how clinical and radiological data can predict this. In our study, we detected a higher death rate among elderly patients with a statistically significant difference and this was a coincidence with Yang et al. [11], Hani et al. [12], Li et al. [13], and Carlos et al. [14]

We detected an association between the presence of multiple multilobar peripheral GGO, white lung, and pleural effusion in CT and the need for mechanical ventilation as the results showed three quarters (75%) of those who showed white lung on the radiological imaging needed ventilation compared with only 6.3% among those without white lung and this association was highly statistically significant, (*p* = <0.001). Half of the patients with pleural effusion experienced shortness of breath or needed ventilation compared with a small percent among those without pleural effusion (7.0 and 8.8%, respectively) and these associations were statistically significant. Also, a significantly higher percentage of patients with pleural effusion (33.3%) suffered from tachypnea compared with only 4.4% among those without (*p* = 0.003). This was matching with Hani et al. [12], Li et al. [15], Peijie et al. [16]

Obviously, tachypnea, shortness of breath, and the requirement of mechanical ventilation were specifically associated with the studied patients with the development of pleural effusion. Peijie et al. [16] and Zhao et al. [17] documented that pleural effusion was much more prevalent in cases who were considered medical emergency than those who were not. The clinical prognosis was poor in cases suffering from tachypnea, shortness of breath, and headache and in the higher age group. Death as an outcome was highly associated with such clinical features. However, tachypnea was the only clinical feature that may be considered a predicting factor for death. So, tachypnea may be considered bad prognostic factors in the COVID-19 disease course.

Limitations of the current study included the retrospective nature of the study in addition to the fact that we only included the findings of the CT at first presentation with the possibility that some patients might present themselves at different stages of the disease; however, we consider this to be a simulation of real-life situation based on different cultural and personal differences between the patients.

## Conclusion

Typical CT findings of GGO showing bilateral distribution with no lobar predilection, crazy-paving consolidation in conjunction with suspecting clinico-laboratory data of COVID-19 are highly diagnostic. We should take into consideration the presence of atypical CT findings when the present does not exclude the presence of COVID-19 infection. CT is considered a prognostic tool as there was the association between the presence of multiple multilobar peripheral GGO, white lung and pleural effusion in CT and the need for ICU admission and mechanical ventilation; this should help us in the referral of patients according to the health-system resources. Regarding mortality, the only significant clinical features that correlated with the patient’s death were age, the presence of shortness of breath, headache, and tachypnea. None of the CT signs including the severe signs of infection in CT correlated with patients’ death; this means that even patients with severe signs of a lung infection on the first presentation have a survival chance.

## Data Availability

The datasets used and/or analyzed during the current study are available from the corresponding author on reasonable request.

## Abbreviations

ARDS: Acute respiratory distress syndrome
COVID-19: Coronavirus disease-19
GGO: Ground-glass opacity
ICU: Intensive care unit
PCR: Polymerase chain reaction
MDCT: Multidetector computed tomography

## References

[1] Anthony R, Stanley P (2015). Coronaviruses: an overview of their replication and pathogenesis. Methods Mol Biol.

[2] Fengxiang S, Nannan S, Fei S, Zhiyong Z, Jie S, Hongzhou L, Yun L, Yebin J, Yuxin S (2020). Emerging 2019 novel coronavirus (2019-nCoV) pneumonia. Radiology.

[3] Adam B, Xueyan M, Mingqian H, Yang Y, Zahi A, Ning Z, Kaiyue D, Bin L, Xiqi Z, Kunwei L, Shaolin L, Hong S, Adam J, Michael C (2020). Chest CT findings in coronavirus disease-19 (COVID-19): relationship to duration of infection.

[4] Zu ZY, Jiang MD, Xu PP, Chen W, Ni QQ, Lu GM, Zhang LJ (2020) Coronavirus disease 2019 (COVID-19): a perspective from China. Radiology.:200490. 10.1148/radiol.2020200490 10.1148/radiol.2020200490 PMC7233368 32083985

[5] Yan L, Liming X (2020). Coronavirus disease 2019 (COVID-19): role of chest CT in diagnosis and management. AJR.

[6] Yoon SH, Lee KH, Kim JY, Lee YK, Ko H, Kim KH, Park CM, Kim YH (2020). Chest radiographic and CT findings of the 2019 novel coronavirus disease (COVID-19): analysis of nine patients treated in Korea. Korean J Radiol.

[7] Tay MZ, Poh CM, Rénia L et al (2020) The trinity of COVID-19: immunity, inflammation and intervention. Nat Rev Immunol. 10.1038/s41577-020-0311-8 10.1038/s41577-020-0311-8 PMC7187672 32346093

[8] Xiaofeng C, Yanyan T, Yongkang M, Shengkai L, Daiying L, Zhijian Y, Zhiqi Y, Hongfu S, Jinming Q, Yuting L, Jianning X, Xiangguang C, Xianheng W, Renhua W, Zhuozhi D A diagnostic model for coronavirus disease 2019 (COVID-19) based on radiological semantic and clinical features: a multi-center study. Eur Radiol. 10.1007/s00330-020-06829-2 10.1007/s00330-020-06829-2 PMC7160614 32300971

[9] Melina H, Soheil K, Ali G, Sravanthi R, Lee M (2020). Radiology perspective of coronavirus disease 2019 (COVID-19): lessons from severe acute respiratory syndrome and Middle East respiratory syndrome. AJR.

[10] Wenjing Y, Arlene S, Xiaochun Z, Guanshu L, Zhongzhao T, Shihua Z, Minjie L The role of imaging in 2019 novel coronavirus pneumonia (COVID-19). Eur Radiol. 10.1007/s00330-020-06827-4

[11] Yang W, Cao Q, Qin L, Wang X, Cheng Z, Pan A (2020). Clinical characteristics and imaging manifestations of the 2019 novel coronavirus disease (COVID-19): a multi-center study in Wenzhou city, Zhejiang, China. J Inf Secur.

[12] Hani C, Trieu NH, Saab I, Dangeard S, Bennani S, Chassagnon G, Revel MP (2020). COVID-19 pneumonia: a review of typical CT findings and differential diagnosis. Diagn Interv Imaging.

[13] Li Q, Guan X, Wu P et al (2020) Early transmission dynamics in Wuhan, China, of novel coronavirus–infected pneumonia. N Engl J Med. 10.1056/NEJMoa2001316 [Epub ahead of print] 10.1056/NEJMoa2001316 PMC7121484 31995857

[14] Carlos W, Dela C, Cao B (2020). Novel Wuhan (2019-nCoV) coronavirus. Am J Respir Crit Care Med.

[15] Li K, Wu J, Wu F, Guo D, Chen L, Fang Z, Chuanming L (2020). The clinical and chest CT features associated with severe and critical COVID-19 pneumonia. Invest Radiol.

[16] Peijie L, Xing L, Rui Z, Lei S, Jianbo G (2020). The performance of chest CT in evaluating the clinical severity of COVID-19 pneumonia: identifying critical cases based on CT characteristics. Invest Radiol.

[17] Zhao W, Zhong Z, Xie X, Yu Q, Liu J (2020) Relation between chest CT findings and clinical conditions of coronavirus disease (COVID-19) pneumonia: a multicenter study. AJR Am J Roentgenol 2020;214:1072–1077 10.2214/AJR.20.22976 32125873

